# Guidelines for Identifying Homologous Recombination Events in Influenza A Virus

**DOI:** 10.1371/journal.pone.0010434

**Published:** 2010-05-03

**Authors:** Maciej F. Boni, Menno D. de Jong, H. Rogier van Doorn, Edward C. Holmes

**Affiliations:** 1 Oxford University Clinical Research Unit, Ho Chi Minh City, Vietnam; 2 MRC Centre for Genomics and Global Health, University of Oxford, Oxford, United Kingdom; 3 Nuffield Department of Medicine, Centre for Tropical Medicine, University of Oxford, Oxford, United Kingdom; 4 Department of Medical Microbiology, Academic Medical Center, University of Amsterdam, Amsterdam, The Netherlands; 5 Department of Biology, Center for Infectious Disease Dynamics, The Pennsylvania State University, State College, Pennsylvania, United States of America; 6 Fogarty International Center, National Institutes of Health, Bethesda, Maryland, United States of America; Institute of Infectious Disease and Molecular Medicine, South Africa

## Abstract

The rapid evolution of influenza viruses occurs both clonally and non-clonally through a variety of genetic mechanisms and selection pressures. The non-clonal evolution of influenza viruses comprises relatively frequent reassortment among gene segments and a more rarely reported process of non-homologous RNA recombination. Homologous RNA recombination within segments has been proposed as a third such mechanism, but to date the evidence for the existence of this process among influenza viruses has been both weak and controversial. As homologous recombination has not yet been demonstrated in the laboratory, supporting evidence, if it exists, may come primarily from patterns of phylogenetic incongruence observed in gene sequence data. Here, we review the necessary criteria related to laboratory procedures and sample handling, bioinformatic analysis, and the known ecology and evolution of influenza viruses that need to be met in order to confirm that a homologous recombination event occurred in the history of a set of sequences. To determine if these criteria have an effect on recombination analysis, we gathered 8307 publicly available full-length sequences of influenza A segments and divided them into those that were sequenced via the National Institutes of Health Influenza Genome Sequencing Project (IGSP) and those that were not. As sample handling and sequencing are executed to a very high standard in the IGSP, these sequences should be less likely to be exposed to contamination by other samples or by laboratory strains, and thus should not exhibit laboratory-generated signals of homologous recombination. Our analysis shows that the IGSP data set contains only two phylogenetically-supported single recombinant sequences and no recombinant clades. In marked contrast, the non-IGSP data show a very large amount of potential recombination. We conclude that the presence of false positive signals in the non-IGSP data is more likely than false negatives in the IGSP data, and that given the evidence to date, homologous recombination seems to play little or no role in the evolution of influenza A viruses.

## Introduction

Influenza A is a rapidly evolving single-stranded negative-sense RNA virus [1]. More than one hundred antigenic subtypes, each containing many variants, are maintained in wild aquatic bird reservoir populations [1]–[3]. Certain subtypes have jumped species and established endemic infections among humans, pigs, horses, and other land and sea mammals [1]. Influenza has shown a propensity to evolve drug resistance [4]–[6], escape immunity [7]–[11], reassort its RNA segments in multiple host types [2], [3], [12]–[17], and possibly but rarely undergo non-homologous recombination in which short regions of sequence are transferred among different segments [18]–[20]. Despite this varied and dynamic life cycle, intra-segment homologous RNA recombination has only been rarely reported in influenza A virus and each of these instances has proven controversial. Some studies have described sequence patterns that seem compatible with homologous recombination events in influenza virus [21]–[23], but refutations of some of these observations have been well argued and convincing [24], [25]. In addition, larger-scale bioinformatic analyses found no evidence for homologous recombination in human influenza A [26] or B [27] virus, and despite a number of exceptions [28], homologous recombination is generally thought to be rare in negative-sense RNA viruses, which may be largely due the presence of a ribonucleoprotein complex that never disassembles from the RNA [29], [30]. However, the shedding of multiple viruses by waterfowl has been reported [2], as has mixed infection of humans [31], providing opportunities for recombination. In addition, recombination may occur during infection with multiple viruses of laboratory cell-lines.

Because of its controversial nature and possible implications for vaccine design, reports of homologous recombination in influenza virus should be carefully examined to exclude all possible alternative hypotheses for the putatively observed recombination event. Some important alternative hypotheses to explore are contamination of samples, false positive bioinformatic signals, and alternative evolutionary histories that generated the apparent recombinants. As a case in point, Krasnitz et al [32] showed that GenBank influenza submissions contain a number of probable contaminants that are labeled with one year but are identical to viruses isolated decades apart, an example being two nearly-identical avian sequences isolated in Taiwan in 1972 and 1987. Similarly, putative recombinants in human H3N2 and H1N1 viral subtypes were isolated decades apart (e.g. 1968 and 2004) from their parental sequences [26]. Given the rapid rate of sequence change in influenza virus, and in particular the rapid rate of viral lineage turnover in human populations, such evolutionary ‘stasis’ seems untenable. A second common pitfall in recombination analysis is failing to exclude the possibility of lineage-specific rate variation. An important case in point concerns the H1N1 influenza A virus associated with the global pandemic of 1918–1919. Although a homologous recombination event between human and swine viruses was proposed in this case [21], a later study showed that the phylogenetic incongruence in this case was entirely due to contrasting patterns of rate variation [25].

Because of the important yet ongoing controversy over the occurrence of homologous RNA recombination in influenza A virus, we herein propose guidelines for how this process can be reliably detected.

## Guidelines

When investigating whether a homologous recombination event could have occurred during the history of a set of sampled influenza viruses, (1) laboratory procedures should be optimized to prevent laboratory-generated artificial recombinants, (2) sequence data should show a statistically significant recombination signal, and (3) the putative recombination event should show plausibility as the most likely hypothesis explaining the data given what is known about the ecology and evolution of influenza viruses.

### Essential Laboratory Procedures

#### 1. Determining if specimens came from co-infected hosts in the absence of recombination

Samples from co-infected hosts will have RNA present from multiple viruses [31], and culture and PCR amplification may result in specific segment sub-regions being amplified from different viruses, giving the appearance of homologous intra-segment recombination where none has occurred. To rule out the possibility that observed recombination is not an artifact of such co-infection, care should be given to amplify and sequence (multiple) clonal virus isolates (biological clones) generated by plaque purification or limiting dilution culture [26]. If sequencing is done directly from clinical specimens (without previous culture), single virus sequencing could be achieved by limiting dilution PCR, although this is more challenging in view of low levels of target RNA. Isolating different viruses from multiple cultures from the same specimen should be the gold-standard method for determining co-infection of a sample. It is important to remember that cross-sample contamination may be an additional mechanism by which multiple viruses are present in culture (see below).

#### 2. Limiting contamination

The risk of contamination during virus isolation can be minimized by the use of clean rooms or, at least, dedicated clean cabinets, for splitting and preparation of cells, to prevent contamination from infected cell cultures, and by meticulous experimental design (e.g. including uninfected wells between different samples when using multi-well plates) and experimental procedure. As described above, sequencing of (multiple) biological clones will guarantee that culture contamination, should it occur, will not result in misinterpretation of possible recombination. During PCR amplification there exist substantial risks of carryover contamination, particularly by previously generated amplicons. Besides meticulous laboratory practice, these risks should be minimized by appropriate laboratory design and workflow for molecular work, including physical separation of rooms for preparation of reagents, nucleic acid extraction, and amplification, respectively, as well as a unidirectional workflow in these respective laboratory rooms. Further minimization of contamination during pre-sequencing PCR reactions could be achieved by commercially available uracil-N-glycosylase-based methods designed to degrade amplicons which may inadvertently be present in samples before PCR amplification. Monitoring of possible PCR contamination by inclusion of sufficient negative controls for extraction and amplification steps remains essential at all times.

### Essential Statistical and Bioinformatic Procedures

#### 3. Assessing statistical significance of phylogenetic recombination signal

The gold-standard bioinformatic approach to demonstrating the presence of recombination is a set of statistically incongruent phylogenetic trees. Because searching for incongruence in trees is extremely laborious when many sequences are present, the preferred pre-screening approach is to scan large data sets for mosaic signals, which are simply incongruent trees with only three sequences included in each tree. Programs such as 3SEQ [33] and Simplot [34] can be readily used to identify potential parents and recombinants; these methods also provide recombination breakpoints and P-values assessing the non-randomness of the mosaic signal. Subsequently, a phylogenetic tree should be inferred including the recombinant(s), all candidate parental sequences, and a collection of other sequences that are representative of the recent and nearby variation in the virus clades under scrutiny. If bootstrap (or similar) support on the tree indicates that the recombinant sequences cluster with one parent group for one sequence region and another parent group for another sequence region, we consider this a statistically-supported phylogenetic recombination signal.

#### 4. Checking sequence alignments for possible errors

Alignment uncertainty can cause discrepancies in phylogenetic inference [35], and possibly in other bioinformatic analyses. Alignments should first be verified to ensure that their lengths correspond to the known segment lengths for influenza A virus. Subsequently, the alignments can be visually analyzed for obvious alignment errors; excluding visually misaligned sequences must be done carefully as true recombinants could also appear to be misaligned.

### Auxiliary Bioinformatic Procedures

#### 5. Determining if breakpoints occur at primer sites

The occurrence of recombination breakpoints at primer sites signifies that different regions of multiple gene segments may have been amplified from a co-infected specimen. A recombination event with inferred breakpoints away from primer sites would ensure that this did not happen. However, this condition is clearly stringent and should be considered carefully as (1) recombination, if it were to occur, could indeed involve breakpoints close to primer sites, and (2) breakpoints can either be reported as ranges (3SEQ) or can vary depending on the choice of a sliding-window size (SimPlot), making it likely that a true recombination breakpoint could result in the identification of a breakpoint range (position) that covers (is close to) a primer site.

### Procedures to Assess Compatibility with Known Ecology and Evolution of Influenza

#### 6. Finding a recombinant clade with sequences isolated from different animals and preferably by different laboratories

Laboratory artifacts for recombination are most likely to be present as single isolates in phylogenetic trees. However, the greater the frequency of this putative recombinant in the circulating virus population, the lesser the probability that it is a false-positive as this would require multiple identical errors to be made; this is particularly true if the putative recombinant is isolated by different laboratories. An important case in point concerns Ebola virus, where an entire recombinant lineage has been identified [28], and which also demonstrate that homologous RNA recombination can indeed occur sporadically in negative-sense RNA viruses. Ideally, parental sequences should also appear as clades and not single sequences to ensure that putative parental sequences did not result from contamination or sequencing error.

#### 7. Assessing compatibility of sampling locations and sampling times

The most obvious example of a posited recombination event defying reasonable patterns of influenza ecology and evolution is one in which two human influenza viruses isolated decades apart are identified as possibly having recombined; the rapid turnover of human influenza virus ensures that viruses isolated more than five or six years apart will never be isolated in the same host. A similar, although rather slower lineage turnover, is also seen in the case of avian [36] and swine [37] viruses. In addition, a recombination event may appear geographically implausible. Equine and swine influenza viruses, for example, both exhibit a strong separation of American and Eurasian strains [16], [38], and recombination events involving parents located on different continents should be treated with caution; the global trade in swine and poultry will make some geographic scenarios more plausible than others [39], [40]. Finally, an obvious signal of a false-positive recombination event is that one of the putative parents is a viral strain that is commonly used in the laboratory. As an example, He et al [22] identified A/Taiwan/4845/99 as a recombinant of A/Wellington/24/2000 and A/WSN/33, the latter of which is a known laboratory strain and previously implicated in contamination events [41]. Clearly, a lineage of naturally occurring influenza virus could not have persisted from 1933 to the 1990s, nor could an ancient putative recombinant lineage survive from 1933 to the 1990s.

#### 8. Determining plausibility of lineage-specific rate variation

As noted previously, lineage-specific rate variation (heterotachy) can produce a mosaic signal identical to one that would be seen under homologous recombination [25], [33]. Specifically, if region A is highly conserved in lineage A, and if region B is highly conserved in lineage B, then any sampled sequence that has evolved in a third separate lineage will appear to be similar to lineage-A sequences in region A and similar to lineage-B sequences in region B, thus appearing to be a mosaic of sequences from lineages A and B. See Boni et al [33] and Worobey et al [25] for examples of how lineage-specific rate variation can cause apparent mosaicism.

### Previously identified homologous recombinants in influenza

We know of only three peer-reviewed articles that have proposed the occurrence of homologous intra-segment recombination in influenza virus [21]–[23].

As described previously, Gibbs et al [21] suggested that the HA segment of the 1918 H1N1 virus was a recombinant between a swine virus and a human virus. However, this claim was strongly refuted by Worobey et al [25], primarily because of a lack of phylogenetic support for the recombination signal and the inability to exclude the alternative hypothesis of lineage-specific rate variation.

He et al [22] present two Canadian swine viruses isolated in 2003, one human H1N1 virus isolated in Taiwan in 1999, and one human H3N2 virus isolated in Hong Kong in 1997 as probable homologous recombinants. One of the Canadian recombination events requires a parental sequence from 1960, and the Taiwanese recombinant requires the laboratory strain A/WSN/33(H1N1) as one of its parents, strongly suggesting that these putative recombination events are erroneous.

The PB2 gene of the A/Swine/Ontario/53518/03 virus has putative parental sequences from 2002 (Korea) and 2003 (Alberta) making it a more plausible recombinant, although it does contain parental strains sampled from different continents. It is identified as a mosaic sequence by 3SEQ (at the same breakpoint) and has a statistically-supported phylogenetic recombination signal in an ML tree constructed by RAxML [42], [43] (analysis not shown). However, in order to infer this tree, all North American and Asian swine H1Nx sequences since 1970 were downloaded from the Influenza Virus Resource, and no other sequence clusters with A/Swine/Ontario/53518/03, making it the lone recombinant out of 181 sequences.

The NP segment of the human H3N2 sequence A/Hong Kong/498/97 has putative parents in Hong Kong (1997 and 1998). This sequence is also identified as a mosaic by 3SEQ (with the same breakpoint), but phylogenetic analysis of the shorter region (223nt) does not produce a statistically-supported clade containing the recombinant and one of the parents (analysis not shown). A total of 517 public H1N1 and H3N2 human sequences were downloaded to infer this tree, and not a single sequence showed the same putative recombination event as A/Hong Kong/498/97; consequently, evidence is lacking for a recombinant clade of viruses like A/Hong Kong/498/97.

The second He et al [23] study presents three avian H9N2 viruses that they sampled, plaque-purified, and sequenced – A/chicken/Guangxi/1/00, A/chicken/Guangxi/14/00, A/chicken/Guangxi/17/00 – and an additional 41 sequences that the authors downloaded from GenBank and analyzed with SimPlot. The three Guangxi viruses appear to have a mosaic PA segment that also has a statistically-supported phylogenetic signal, and which therefore constitute the closest example we have seen thus far of a recombinant clade, although it is noteworthy that they were obtained in the same laboratory. Here, investigating guidelines 2 and 5 further would give us some certainty as to the veracity of these recombinant sequences. Indeed, it is important to note that the parental sequences for these three putative recombinants were identified in GenBank, and their sample-handling and sequencing are of unknown quality.

The remaining 41 recombinants are shown with phylogenies and SimPlot graphs in the supplementary materials (Figures S1, S2, S3, S4, S5, S6, S7, S8, S9, S10, S11, S12, S13, S14, S15, S16, S17, S18, S19, S20, S21, S22, S23, S24, S25, S26, S27, S28, S29, S30, S31, S32, S33, S34, S35, S36, S37, S38 in the He et al paper). These are mainly H5N1 and H9N2 chicken isolates from China, and 31 of the recombination events occur in the polymerase genes, which are the longest segments with the most number of individual contigs. As we show in the next section, downloading sequences from GenBank without controlling for quality can lead to the inclusion of many sequences from contaminated samples which can generate false-positive recombination signals.

### Controlling for quality in the influenza sequence database

To provide some control for quality of sequencing, we can separate the database of all publicly available influenza sequences into those that were sequenced by the Influenza Genome Sequencing Project and those that were not. Importantly, sequence data generated under the IGSP are subject to very strict quality control, manifest as a high level of redundancy. Specifically, the sequencing procedure utilizes both short amplicons and overlapping amplicons, so that each nucleotide is covered by at least two separate amplicons and each amplicon is sequenced at least twice in both the forward and reverse directions [44].

To assess the possible effect of sequencing quality on detection of recombination signals, we performed a recombinant search on IGSP and non-IGSP avian influenza viruses, isolated from birds and humans, similar to the search and analysis in Boni et al [26]. Identification of recombinants is done according to the guidelines defined in this paper.

## Methods

All influenza sequences were downloaded from the Influenza Virus Resource (http://www.ncbi.nlm.nih.gov/genomes/FLU/Database/select.cgi) on March 18–20, 2009, excluding seasonal human H3N2 and seasonal human H1N1 as these were analyzed elsewhere [26]. Data sets that had a sizeable contribution (>30) of both IGSP sequences and non-IGSP sequences were aligned with MUSCLE v3.6 [45] and analyzed for recombination signals using the 3SEQ algorithm [33], [46]. 3SEQ tests all possible triplets in a data set for a mosaic recombination signal using a nonparametric statistic for mosaicism. 3SEQ also infers breakpoints and reports likely breakpoint ranges.

MUSCLE alignments were inspected visually to remove sequences that were obvious alignment errors. Hence, GenBank accession number DQ997359 (non-IGSP) was removed from the avian/H5N1/PA data set, while GenBank accession number DQ997548 (non-IGSP) was removed from the avian/H5N1/NA data set. Three IGSP sequences (CY029003, CY015123, CY031005) and 14 non-IGSP sequences (AF144307, AY059499, AF216718, AF216726, AF216734, AF216710, AY059502, AY059503, AY059501, FJ434376, EU636696, U85447, U85380, AY028445) were removed from the avian/H5N1/NS data set. Two IGSP sequences (CY005144, CY005877) and 11 non-IGSP sequences (AF156485, EU982320, AF156484, EU982312, AY633280, AF508712, AF523504, AB256686, AB256718, AF523503, AF523505) were removed from the avian/H9N2/NS data set. Removing these misaligned sequences had no effect on the IGSP recombination analysis and inconsequential effects on the non-IGSP analysis.

Candidate recombinant sequences were identified as having a corrected P-value<0.05 as determined by 3SEQ's nonparametric Δ-statistic [33]. All candidate recombinants were then filtered to retain only those where both putative recombinant regions were longer than 100nt, so that phylogenetic trees could be reasonably inferred for both regions. Phylogenetic incongruence was again utilized as the gold-standard bioinformatic method for the identification of recombinant sequences. Maximum likelihood (ML) trees were inferred with PAUP* [47] and RAxML [42], [43]; bootstrap analysis was performed with RAxML. A total of 6110 non-IGSP sequences and 2197 IGSP sequences were tested for homologous recombination with 3SEQ by testing for recombination signals in all possible sequence triplets within each of the 18 data sets described in Table 1.

**Table 1:** Summary of 18 datasets used in this study.

			number of sequences		segregating sites		Watterson's è		P-value		number of recombinant sequences		number of recombinant sequences >100nt		number of bootstrap-supported phylogenetic recombination signals
host	subtype	segment	non-IGSP	IGSP	non-IGSP	IGSP	non-IGSP	IGSP	non-IGSP	IGSP	non-IGSP	IGSP	non-IGSP	IGSP	IGSP
avian	H5N1	PB2	402	114	1279	844	0.089	0.074	0	1.63×10^−3^	387	1	382	0	0
		PB1	374	219	1112	870	0.079	0.068	10^−29^	2.87×10^−5^	337	182	335	0	0
		PA	494	227	1226	834	0.085	0.066	10^−36^	1.04×10^−5^	465	16	465	16	1
		HA	1155	230	1218	759	0.095	0.077	10^−8^	0.00278	101	1	6	0	0
		NP	495	200	759	494	0.077	0.058	10^−25^	0.00265	423	1	95	0	0
		NA	829	222	951	614	0.093	0.075	10^−10^	6.52×10^−6^	201	9	67	0	0
		MP	440	116	466	237	0.068	0.044	10^−6^	0.313	72	0	54	0	0
		NS	566	193	530	298	0.092	0.063	10^−7^	1	335	0	190	0	0
human	H5N1	PB2	55	42	606	202	0.064	0.023	3.24×10^−5^	0.323	4	0	3	0	0
		PB1	62	41	501	196	0.049	0.021	0.0108	1	3	0	3	0	0
		PA	65	44	563	179	0.06	0.021	2.96×10^−5^	1	7	0	0	0	0
		HA	142	48	581	185	0.065	0.026	0.0254	1	3	0	0	0	0
		NP	60	43	297	107	0.046	0.018	10^−7^	1	7	0	0	0	0
		NA	151	41	564	137	0.075	0.025	10^−8^	1	106	0	54	0	0
		MP	63	40	189	75	0.044	0.02	8.04×10^−5^	0.999	13	0	13	0	0
		NS	67	33	199	60	0.052	0.019	0.974	1	0	0	0	0	0
avian	H9N2	NA	377	190	966	658	0.109	0.084	10^−16^	10^−10^	159	39	119	33	1
		NS	313	154	498	352	0.095	0.077	0.628	1	0	0	0	0	0

**Footnotes:** P-values are corrected with a Dunn-Sidak correction for the numbers of triplets tested in each dataset. P-values are shown as simple orders of magnitude when P<10^−6^.

To test if the strong recombination signals detected in the non-IGSP data sets could result from false positive identification by the 3SEQ algorithm, 3SEQ was run on computer-generated clonal data with the numbers of sequences, models of evolution, and diversity parameters mimicking those of the non-IGSP data sets. Models of evolution were evaluated using MODELTEST [48]; for each sequence set, GTR+Γ was either the best model or within the 95% confidence bounds given by the Akaike Information Criterion. For each of the 18 non-IGSP data sets, 100 clonal data sets – of the same size, diversity, and evolved under the same GTR+Γ model – were generated using Treevolve [49]. These 18×100 clonal data sets were analyzed by 3SEQ for recombination signals; results are in Table 2.

**Table 2:** False positives of 3SEQ algorithm on simulated clonal data sets of the same size and diversity as in this study.

host	subtype	segment	number of 3SEQ false positives using P<.05	number of 3SEQ false positives using P<.50
human	H5N1	PB2	1 / 100	3 / 100
	H5N1	PB1	0 / 100	0 / 100
	H5N1	PA	0 / 100	1 / 100
	H5N1	HA	0 / 100	1 / 100
	H5N1	NP	0 / 100	3 / 100
	H5N1	NA	0 / 100	0 / 100
	H5N1	MP	1 / 100	2 / 100
	H5N1	NS	0 / 100	1 / 100
avian	H5N1	PB2	0 / 100	1 / 100
	H5N1	PB1	0 / 100	0 / 100
	H5N1	PA	0 / 100	1 / 100
	H5N1	HA	0 / 100	0 / 100
	H5N1	NP	0 / 100	1 / 100
	H5N1	NA	0 / 100	0 / 100
	H5N1	MP	0 / 100	0 / 100
	H5N1	NS	0 / 100	0 / 100
avian	H9N2	NA	0 / 100	0 / 100
	H9N2	NS	0 / 100	1 / 100

**Footnote:** P-value in the column heading is the Dunn-Sidak corrected P-value.

## Results

A total of 1786 (29%) non-IGSP sequences had recombination regions of sufficient length (>100nt) to be tested by phylogenetic inference, while only 49 (2%) of IGSP sequences had such signals. In the non-IGSP set, 31% of all avian sequences had putative recombinant regions >100nt, while 11% of all human sequences had putative recombinant regions >100nt. Sixteen of the putative IGSP recombinants were avian H5N1 sequences of the influenza PA gene segment (acidic subunit of the RNA polymerase). The remaining 33 were avian neuraminidase (NA) sequences of the H9N2 subtype. These 49 sequences represent 2.6% of all avian IGSP sequences. The putative IGSP recombinants are considered in detail below.

For the PA gene, the breakpoint pair 1361/1938 resulted in one candidate recombinant (A/Muscovy Duck/Vietnam/NCVD-22/2007), and the breakpoint pair 139/2015 corresponded to the other 15 putative recombinants. The pair 139/2104 was also considered but did not show any phylogenetic recombination signal (results not shown).

**Figure 1:**
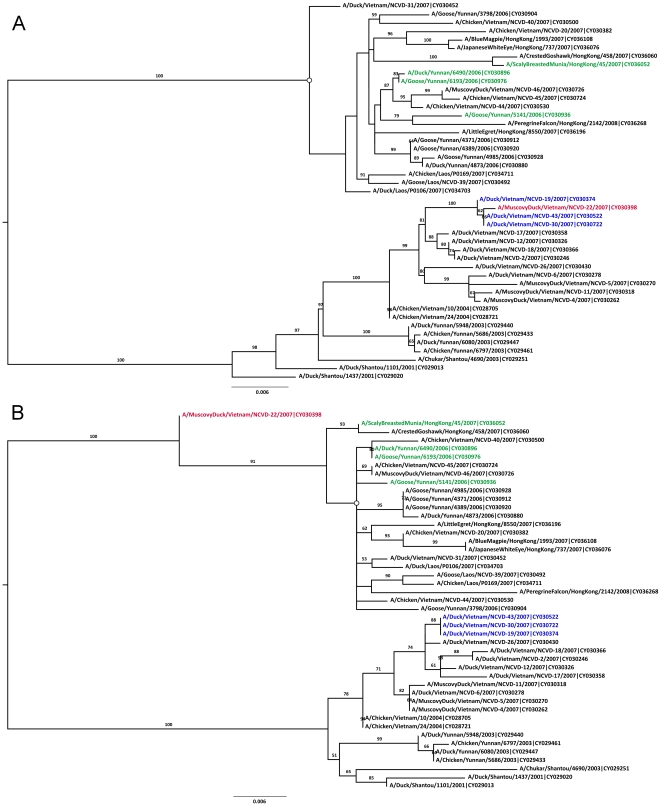
Bootstrapped ML trees, inferred with RAxML, for regions of the PA segment of avian H5N1 sequences. Tree A was inferred for the region defined by positions 1–1361 & 1939–2314. Tree B was inferred for the region defined by positions 1362–1938. The red sequence is the putative recombinant sequence. Blue and green sequences are major and minor parental sequences, respectively, as identified by 3SEQ. Trees are midpoint rooted. ML trees inferred with PAUP* have some non-critical differences the two subclades marked with open circles.

The inferred phylogenies of the ‘major’ (bases 1–1361 and 1939–2314) and ‘minor’ (bases 1362–1938) regions of the avian H5N1 PA gene are shown in Figure 1. The blue and green colored sequences in these figures were identified as the major and minor parental sequences, respectively, by 3SEQ. The putative recombinant A/Muscovy Duck/Vietnam/NCVD-22/2007 appears to jump clades in these two phylogenies, and its clustering with the blue clade in one tree and the green clade in the second tree is supported by bootstrap percentages of 100 and 91, respectively, making this a well-supported phylogenetic recombination signal.

**Figure 2:**
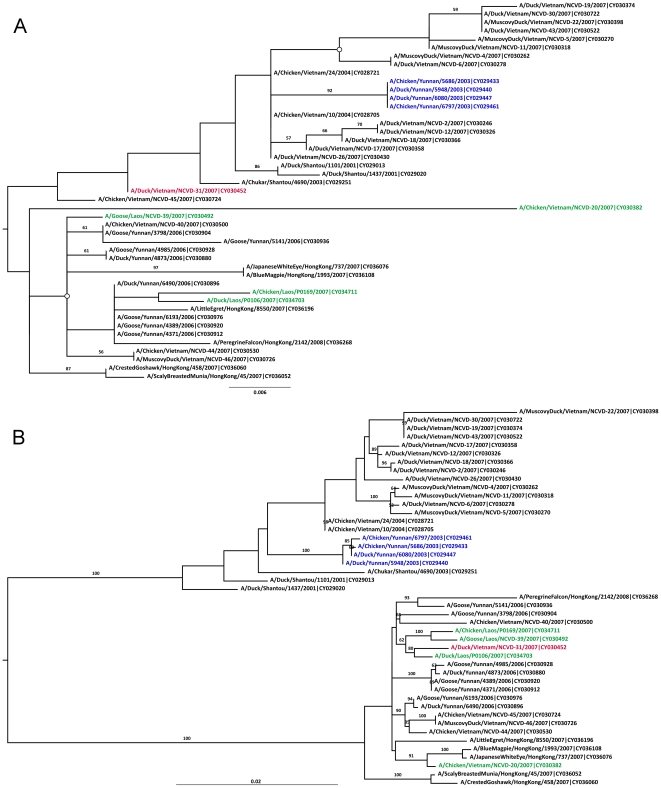
Bootstrapped ML trees for the PA segment of avian H5N1 sequences. Tree A was inferred for the region defined by positions 1–139 & 2016–2314. Tree B was inferred for the region defined by positions 140–2015. ML trees inferred with PAUP* have some non-critical differences the two subclades marked with open circles. Phylogenetic relationships in these trees do not support a hypothesis of homologous recombination. Other features as in Figure 1.

Figure 2 shows the same 45 sequences as Figure 1, but with the major region now defined as bases 1–139 and 2016–2314, and the minor region as bases 140–2015. Fifteen sequences were identified by 3SEQ as having mosaic signals that corresponded to these breakpoints, but only one sequence (A/Duck/Vietnam/NCVD-31/2007) exhibits phylogenetic incongruence in these trees. Unfortunately, the major region is relatively short (438nt), and a robust tree cannot be easily inferred. Low levels of bootstrap support and the putative recombinant not clustering with the parental sequences in Figure 2A indicate that we have little compelling evidence supporting the PA segment of A/Duck/Vietnam/NCVD-31/2007 as a true homologous recombinant.

**Figure 3:**
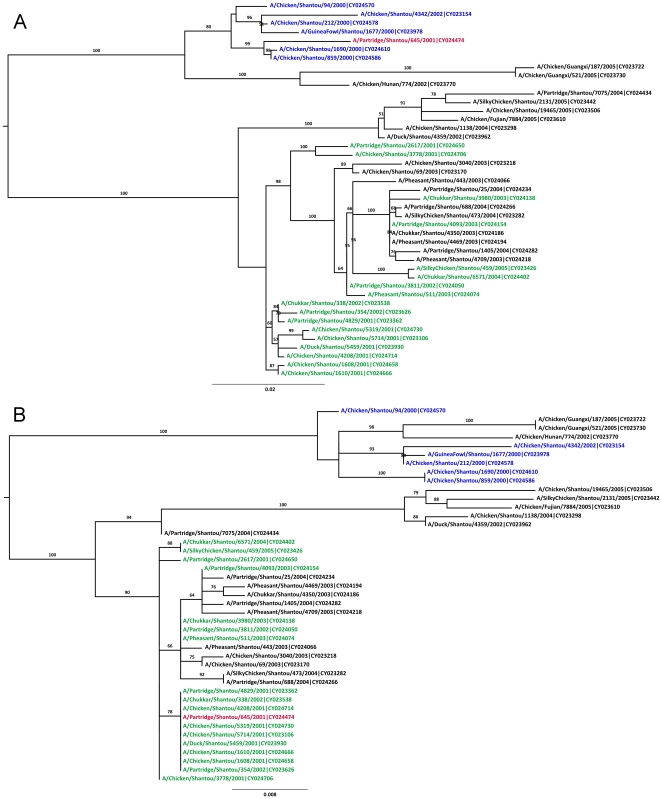
Bootstrapped ML trees for the NA segment of avian H9N2 sequences. Tree A was inferred for the region defined by positions 1–479 & 926–1496. Tree B was inferred for the region defined by positions 480–925. ML trees inferred with PAUP* have identical topology to the trees shown here. Other features as in Figure 1.

For the NA gene, the breakpoint pair 479/925 corresponded to 32 candidate recombinants. The twelve sequences with the strongest signals are included in the trees in Figure 3; a full analysis with all 32 candidate recombinants only showed one (A/Partridge/Shantou/645/2001) with a phylogenetically supported recombination signal. A large number of candidate major parents (6) and minor parents (17) results in a very pronounced phylogenetic recombination signal in Figure 3. High levels of bootstrap support (≥90) on various branches separating the recombinant from the parents suggest that the phylogenetic recombination signal for A/Partridge/Shantou/645/2001 is statistically significant.

**Figure 4:**
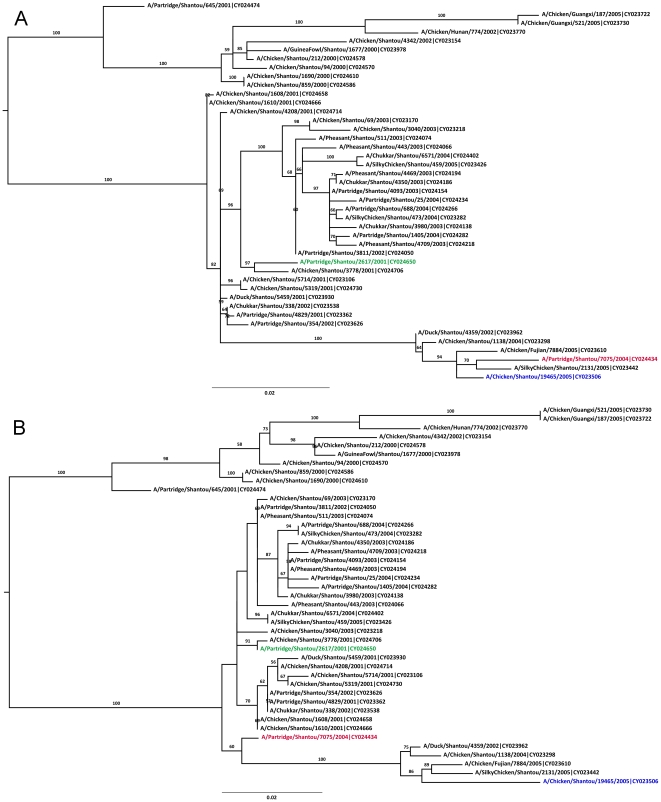
Bootstrapped ML trees for the NA segment of avian H9N2 sequences. Tree A was inferred for the region defined by positions 1–219 & 721–1496. Tree B was inferred for the region defined by positions 220–720. ML trees inferred with PAUP* have identical topology to the trees shown here. Phylogenetic relationships in these trees do not support a hypothesis of homologous recombination. Other features as in Figure 1.

The breakpoint pair 219/720 corresponded to one putative recombinant (A/Partridge/Shantou/7075/2004) shown in Figure 4. The tree inferred for the minor segment (Figure 4A) supports a small degree of phylogenetic incongruence, but not indicative of homologous recombination of the given parental sequences, or any other sequences in this tree.

In summary, of these four recombination scenarios, two had phylogenetically supported recombination signals (Figures 1 and 3); however, in both of these cases, we see only a single circulating recombinant sequence such that they cannot be assigned with any certainty.

Because of the large number of recombination signals in the non-IGSP data, there was no sensible way to choose a subset for phylogenetic analysis. The signals detected in the non-IGSP data represent either (*i*) true recombination, (*ii*) false-positive recombination signals resulting from sequencing error or sample contamination, or (*iii*) false positives generated by the 3SEQ algorithm. To eliminate the last of these three hypotheses, we tested if 3SEQ has an unusually high rate of false positives on data sets with parameters similar to the examined non-IGSP data by simulating 100 clonal versions of each of the 18 non-IGSP data sets used in this study. Table 2 shows that false positive signals would not be expected in such data sets when they are truly clonal, even if the multiple comparisons correction (Dunn-Sidak) in 3SEQ were relaxed by an order of magnitude.

## Discussion

Of the 2197 IGSP sequences analyzed in this study, only two had both a statistically significant mosaic signal and bootstrap-supported recombination signal: the PA segment of A/Muscovy Duck/Vietnam/NCVD-22/2007 (H5N1) and the NA segment of A/Partridge/Shantou/645/2001 (H9N2). While the recombination signals for these sequences seem relatively strong, it is telling that no other samples (IGSP or non-IGSP) had sequences similar at the nucleotide level to these two apparent recombinants. This can mean one of a number of things: (*i*) that homologous recombination is extremely rare, (*ii*) that homologous recombination produces non-viable viruses which cannot be sampled, or (*iii*) that these samples are not in fact true recombinants. Identification of a circulating clade of recombinant viruses, rather than a lone recombinant sequence, would provide far more compelling evidence that intra-segmental homologous recombination occurs among influenza viruses.

If we assume that the sequences generated through the Influenza Genome Sequencing Project are less likely to be contaminated or to contain fewer sequencing errors because of the rigorous quality control used, then the large number of recombination signals present in the non-IGSP data may not reflect the true evolutionary history of these sequences, especially when we note that for some of the longer segments (PB2, PB1, PA), more than 90–95% of non-IGSP sequences were flagged as recombinant. As viruses sequenced through the IGSP should not recombine more or less frequently than viruses sequenced otherwise, either the non-IGSP recombinants are false positives or we have failed to identify true instances of recombination in the IGSP data sets. As 3SEQ has very high power to detect recombination signals [33], especially in data sets with high nucleotide diversity, it is unlikely that we missed scores or hundreds of recombination signals in the 2197 IGSP sequences considered. The more likely scenario is that the non-IGSP signals are false positives.

It is therefore our belief that the true level of homologous recombination is likely to be overestimated in bioinformatic analyses that broadly include all influenza data available in GenBank, especially for avian viruses since birds appear to be very commonly co-infected [2]. It is not true that non-IGSP GenBank sequences must necessarily be low quality, as the large majority of these sequences probably have no sequencing error and come from laboratories where samples are handled very carefully. Rather, the large number of candidate recombinants present in the non-IGSP columns of Table 1 reflects the fact that one recombinant sequence can generate many mosaic signals as it can also be identified as a major or minor parent in the analysis. In the avian PB1 data set, for example, 41 sequences (11%) can be removed and the recombination signal for PB1 disappears altogether, indicating that it is a minority of problematic sequences responsible for the strong recombination signals in the non-IGSP data sets.

As the influenza virus sequence database grows — especially in the next few years as the initial evolution of 2009 H1N1 is tracked — and as advances in computing power and bioinformatic methods are able to handle larger volumes of sequence data, it is clearly of utmost importance not to lose track of sequence quality and sample quality as important prerequisites for the validity of analyses carried out on large data sets.
